# Effect of Twice-Daily Blue Light Treatment on Matrix-Rich Biofilm Development

**DOI:** 10.1371/journal.pone.0131941

**Published:** 2015-07-31

**Authors:** Denise Lins de Sousa, Ramille Araújo Lima, Iriana Carla Zanin, Marlise I. Klein, Malvin N. Janal, Simone Duarte

**Affiliations:** 1 Department of Dental Clinics, School of Pharmacy, Dentistry and Nursing, Federal University of Ceara, Fortaleza, Ceara, Brazil; 2 Department of Biomaterials, State University of São Paulo, Araraquara, São Paulo, Brazil; 3 Department of Epidemiology and Health Promotion, College of Dentistry, New York University, NYU, New York, United States of America; 4 Department of Basic Science and Craniofacial Biology, College of Dentistry, New York University, New York, United States of America; The Scripps Research Institute and Sorrento Therapeutics, Inc., UNITED STATES

## Abstract

**Background:**

The use of blue light has been proposed as a direct means of affecting local bacterial infections, however the use of blue light without a photosensitizer to prevent the biofilm development has not yet been explored. The aim of this study was to determine how the twice-daily treatment with blue light affects the development and composition of a matrix-rich biofilm.

**Methodology/Principal Findings:**

Biofilms of *Streptococcus mutans* UA159 were formed on saliva-coated hydroxyapatite discs for 5 days. The biofilms were exposed twice-daily to non-coherent blue light (LumaCare; 420 nm) without a photosensitizer. The distance between the light and the sample was 1.0 cm; energy density of 72 J cm^-2^; and exposure time of 12 min 56 s. Positive and negative controls were twice-daily 0.12% chlorhexidine (CHX) and 0.89% NaCl, respectively. Biofilms were analyzed for bacterial viability, dry-weight, and extra (EPS-insoluble and soluble) and intracellular (IPS) polysaccharides. Variable pressure scanning electron microscopy and confocal scanning laser microscopy were used to check biofilm morphology and bacterial viability, respectively. When biofilms were exposed to twice-daily blue light, EPS-insoluble was reduced significantly more than in either control group (CHX and 0.89% NaCl). Bacterial viability and dry weight were also reduced relative to the negative control (0.89% NaCl) when the biofilms were treated with twice-daily blue light. Different morphology was also visible when the biofilms were treated with blue light.

**Conclusions:**

Twice-daily treatment with blue light without a photosensitizer is a promising mechanism for the inhibition of matrix-rich biofilm development.

## Introduction

*Streptococcus mutans* is known as the major etiological agent in dental caries [1] and it has a great influence on the development and composition of pathogenic biofilms mainly due to its ability to synthesize extracellular polysaccharides (EPS) [2]. EPS play a major role in the pathogenesis of dental caries, by promoting bacterial accumulation to the tooth surface and influencing the physical and biochemical properties of biofilms [3]. Bacteria embedded in biofilm are significantly less sensitive to different antibacterial agents than free-floating planktonic bacterial cells. It is assumed that bacteria in biofilm express genes that allow for better acclimation to the biofilm microenvironment and contribute to limited diffusion of the bacteria through the biofilm [4,5]. The analyses of EPS matrix formation could advance the current understanding of the development process and structural organization of oral biofilms, which would be essential for designing novel and effective antibiofilm therapies [6].

The photodynamic antimicrobial chemotherapy (PACT) has been indicated as an alternative to conventional antimicrobial therapy to kill oral bacteria [5,7,8]. It is based on the use of extrinsic photosensitizers (PS), light-absorbing molecules that initiate a photochemical reaction when exposed to light of a specific wavelength. This photochemistry process leads to reactive oxygen species (ROS) formation, which may cause irreversible damage to essential bacterial cell compounds and change cell metabolism resulting in bacterial death [9]. The greatest PACT limitation is the challenge for the PS to penetrate through the depths of the biofilm. The phototherapy with blue light (400–500 nm wavelength) without PS seems to be a promising alternative for PACT since it exceeds this challenge. Its antimicrobial mechanism is similar to PACT, however, the bacterial killing seems to involve the activation of endogenous PS in bacteria, such as flavins and cytochromes, that may lead to production of ROS [10].

The antimicrobial effect of blue light alone has been demonstrated in planktonic cultures [10–13]; however, its effect in biofilms has been poorly investigated [12,14]. The blue light’s mechanism of action on the *S*. *mutans* biofilm and how it affects its biofilm structural organization has still not been found. Therefore, the aim of this study was to determine how the daily treatment with blue light affects the development and composition of a matrix-rich *S*. *mutans* biofilm.

## Materials and Methods

### Light Sources

The light source used in this research was a non-coherent, commercially available, blue light (LumaCare Model LC-122, Medical Group, Newport Beach, CA) with 420 nm wavelength, spot size of 113.1 mm^2^, and fixed output power of 95.5 mW cm^-2^.

### Inoculum and biofilm model

*Streptococcus mutans* UA159 (ATCC 700610) was obtained from single colonies isolated on agar plates, inoculated in tryptone yeast-extract broth containing 1% (w/v) glucose and incubated for 18–24 h at 37°C under microaerophilic conditions (5% of CO2). Biofilms of *S*. *mutans* UA159 were formed on saliva-coated hydroxyapatite discs (HA) (0.635 cm^2^) placed in 2 mL of medium containing 1% sucrose, in 24-well cell culture plates, at 37°C, 5% CO_2_, during 5 days. Medium was replaced once daily [15]. Saliva-coated discs were prepared through incubation with de-identified clarified human whole saliva for 1 hour at 37°C in a 3-D rotator (LabLine—Thermo scientific, USA) as previously described [15]. Adsorption buffer (50 mM KCl, 1.0 mM KPO4, 1.0 mM CaCl, 0.1 mM MgCl2 –pH = 6.5–1:1) was added to the saliva as well as phenylmethylsulfonyl fluoride (PMSF– 1:1000). The solution was clarified by centrifugation at 8,500 rpm for 10 minutes at 4°C, filtered with a 0.22 μm pore size filter (Stericup, Millipore, USA) and immediately used [15]. As no identifying information about the donor was collected, this research does not meet the definition of human subjects 45 CFR 46.102(f) and does not require IRB oversight. The biofilms were grown in tryptone yeast-extract broth containing 1% (w/v) sucrose (2 mL/well) and were kept undisturbed for 24 h to allow initial biofilm formation. The culture medium was replaced once daily.

### Exposure of the biofilm to blue light

After initial biofilm formation, the biofilm was exposed to non-coherent blue light [16] twice daily (10 a.m. and 4 p.m.) until the fifth day of the experimental period. The distance between the light source tip and the exposed sample was 1.0 cm and the parameters adopted were energy density of 72 J cm^-2^ and time exposure of 12 min 56 s. Positive and negative control groups were treated twice-daily with 0.12% chlorhexidine—CHX (1min) and 0.89% NaCl (1min), respectively.

### Biofilm analysis

At the end of the experimental period, the biofilms were dip-washed three times, placed in 5 ml sterile saline solution, and the hydroxyapatite surfaces were gently scraped with a sterile spatula to harvest adherent cells. The removed biofilms were subjected to sonication using three 15-s pulses at an output of 7 W (Fisher Scientific, Sonic Dismembrator model 100; USA). The homogenized suspension was used for dry weight, bacterial viability (colony forming units—CFU mg^-1^ of biofilm dry weight), and polysaccharide analyses (EPS-soluble, EPS-insoluble and intracellular polysaccharides—IPS) as previously described [17].

### Dry weight

For the dry weight determination, three volumes of cold ethanol (-20°C) were added to 1 ml biofilm suspension, and the resulting precipitate was centrifuged (10,000 g for 10 min at 4°C). The supernatant was discarded, and the pellet was washed with cold ethanol, and then lyophilized and weighed [17].

### Bacterial viability

An aliquot (0.1 mL) of the homogenized suspension was serially diluted (1:10, 1:100, 1:1000, 1:10000, 1:100000, 1:1000000) and plated on blood agar. The plates were incubated in 5% CO_2_ at 37°C for 48 h, and then the number of CFU mg^-1^ of biofilm dry weight were determined [15].

### Polysaccharide analyses

Soluble and insoluble extracellular polysaccharides (EPS-soluble and EPS-insoluble, respectively), and intracellular polysaccharides (IPS) were analyzed as described previously [17]. Briefly, the polysaccharide content was expressed per mg of polysaccharide by dry weight of total biofilm. Briefly, an aliquot (3.9 ml) of the suspension was sonicated for 30 s pulses at an output of 7 W and centrifuged at 10,000 g for 10 min at 4°C. The supernatant was collected and the biofilm pellet was resuspended and washed in 5 ml of milli-Q water; this procedure was repeated three times. The supernatant was used for the EPS-soluble assay and biofilm pellet was used for the EPS-insoluble and IPS assays. All of the supernatants were pooled and three volumes of cold ethanol were added, and the resulting precipitate was collected by centrifugation and resuspended in 5 ml Milli-Q water; the total amount of carbohydrate was determined by the phenol—sulfuric acid method [18]. The EPS-insoluble was extracted using 1 N NaOH (1 mg biofilm dry weight/0.3 ml of 1 N NaOH) under agitation for 1 h 10 min at 37°C. The supernatant was collected by centrifugation, and the precipitate was resuspended again in 1 N NaOH; this procedure was repeated three times. The total amount of carbohydrate was determined by phenol—sulfuric acid colorimetric method [18]. The IPS were extracted with hot 5.3 M KOH (0.8 mg of biofilm dry weight/ml KOH) and 5.3 M HCl (0.8 mg of biofilm dry weight/ml HCl), and quantified using 0.2% I_2_/2% KI solution and 1 M Phosphate Buffer (pH 7.0) [19].

### Variable pressure scanning electron microscopy (VPSEM)

The HA discs were transferred to glass slides, biofilms upwards, and placed on the VPSEM [Zeiss EVO 50 (Carl Zeiss Microscopy, LLC, Thornwood, NY)] chamber. The images were captured at 100 Pa and 15.00 Kv and working distance was of 7.5 mm [20].

### Confocal scanning laser microscope (CSLM)

The organization of the live and dead bacteria on the biofilm surface was examined by confocal scanning laser microscope. Leica TCS SP5 microscope (Leica Lasertechnik GmbH, Heidelberg, Germany) with a HCX APOL U-V-I 40X/0.8-numerical-aperture water immersion objective was used. The biofilms treated with blue light and control samples were stained with a live/dead BacLight bacterial viability kit (Molecular Probes. Invitrogen, Eugene, Oregon. USA). The stains were prepared in accordance with the manufacturer. The microplates were incubated at room temperature in the dark for 15 min and examined under a CSLM [21]. The bacterial biomass (μm^3^/μm^2^) was quantified using COMSTAT [22]

### Statistical analyses

Prior to analysis, assumptions of equality of variances and normal distribution of errors were checked. As the between group variances were heterogeneous, data were rank transformed prior to analysis with a mixed model ANOVA with a fixed factor of group and a random intercept. When significant differences were detected, the groups were compared using t-tests based on the pooled standard error. IBM SPSS version 21 (IBM, Inc., Armonk, NY) was used to perform the analyses. The confidence interval was set at 95%.

## Results

**Fig 1:**
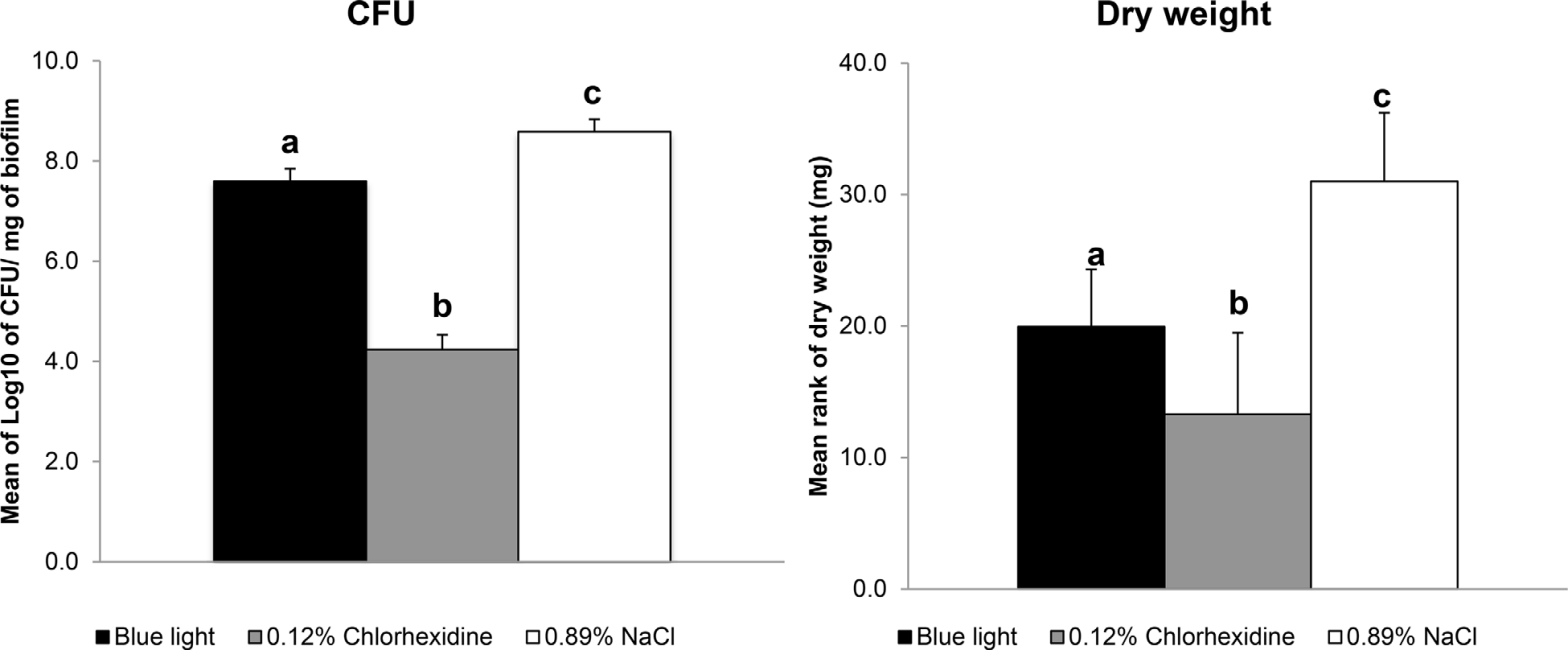
CFU count and dry weight in *S*. *mutans* biofilm. CFU count and dry weight in the *S*. *mutans* biofilm after the twice-daily blue light treatment compared with twice daily treatment with 0.12% Chlorhexidine (positive control) and twice-daily treatment with 0.89% NaCl (negative control). Data represent the mean values and error bars represent standard deviations. Values marked by the different letters are significantly different from each other (p < 0.05).

Fig 1 shows that twice-daily blue light exposure produced intermediate levels of *S*. *mutans* biofilm weight; less than the negative control group treated with 0.89% NaCl (p< 0.05), but more than the positive control group treated with CHX (p< 0.05). CFU counts were reduced only in the positive control group treated with CHX (p< 0.05).

**Fig 2:**
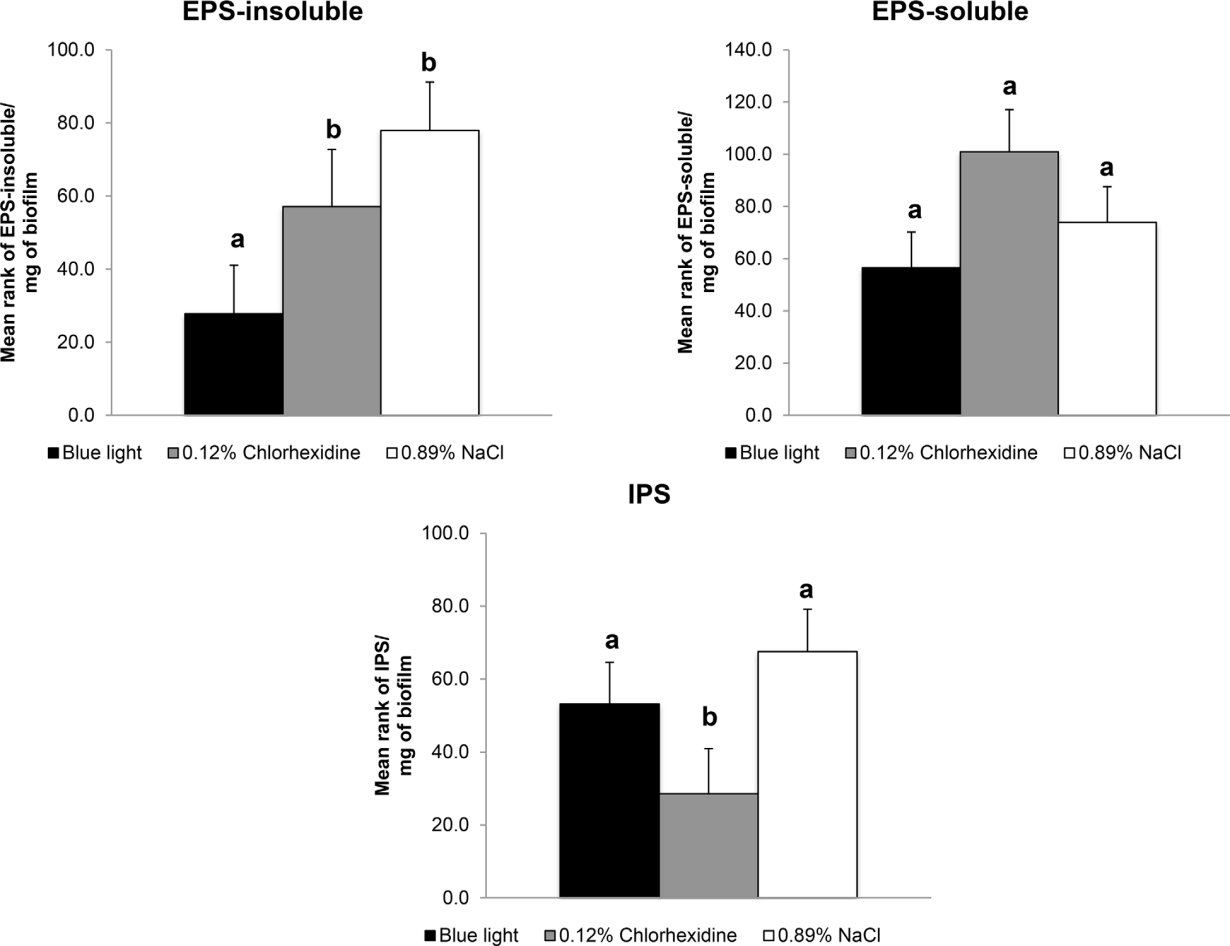
The content of EPS-soluble, EPS-insoluble and IPS in *S*. *mutans* biofilm. The content of soluble and insoluble extracellular polysaccharides (EPS-soluble and EPS-insoluble, respectively), and of intracellular polysaccharides (IPS) in *S*. *mutans* biofilm (expressed in μg/mg of biofilm) after the twice-daily blue light treatment compared with twice daily treatment with 0.12% Chlorhexidine (positive control) and twice-daily treatment with 0.89% NaCl (negative control). Data represent the mean values and error bars represent standard deviations. Values marked by the different letters are significantly different from each other (p < 0.05).

Fig 2 shows that twice-daily blue light exposure produced a larger reduction of EPS-insoluble than in either control group, the positive treated with CHX (p< 0.05) and negative treated with 0.89% NaCl. EPS-soluble levels were unaffected by any treatment and IPS were reduced only in the group treated with CHX (p< 0.05).

**Fig 3:**
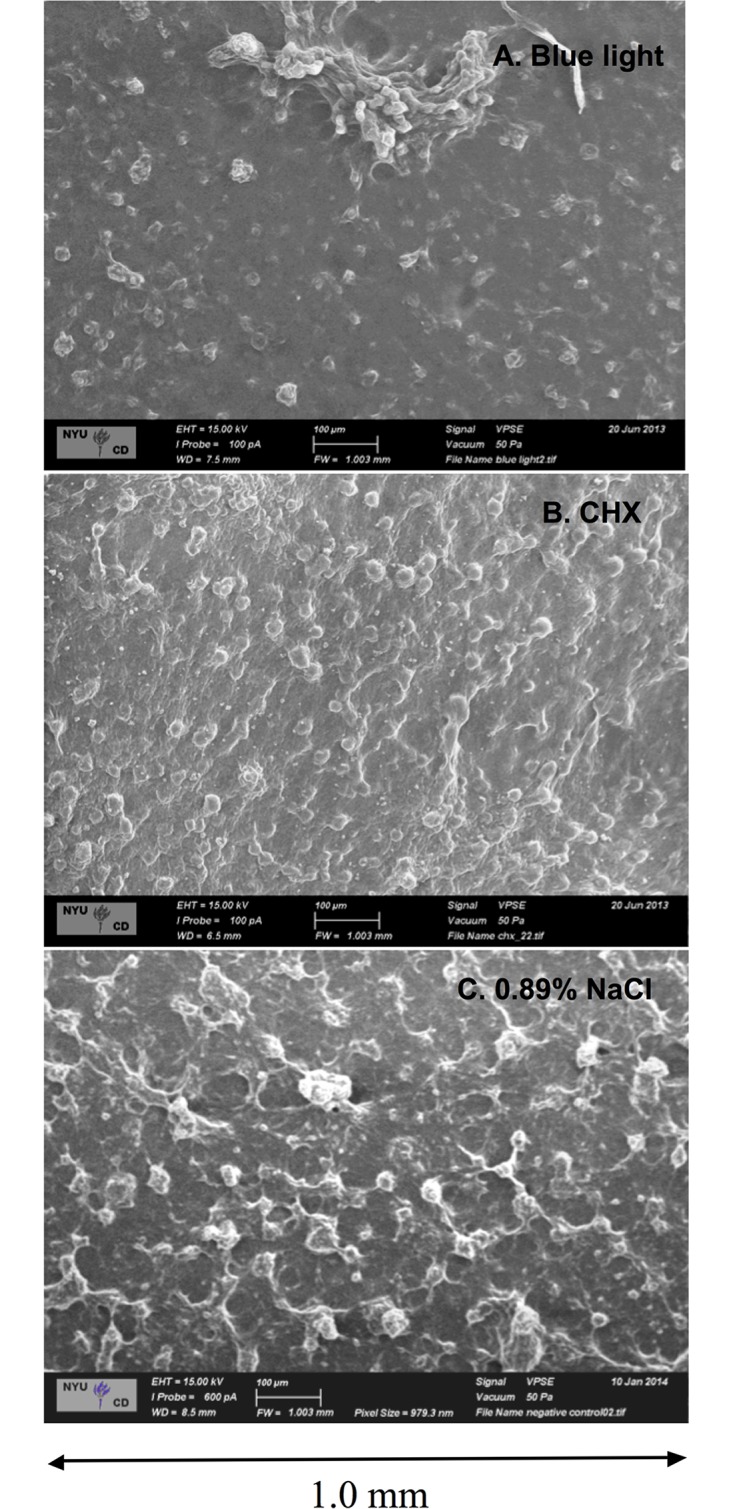
Morphology and structure of *S*. *mutans* biofilms imaged by VPSEM. VPSEM images are showing the morphology and structure of *S*. *mutans* biofilm after the twice-daily blue light treatment compared with positive and negative controls [Field width of 1.0 mm]. A = Biofilm after the twice daily blue light treatment; B = Biofilm after treatment with 0.12% chlorhexidine (positive control); C = Biofilm after treatment with 0.89% NaCl (negative control).

The VPSEM images illustrate the effects of blue light on the morphology and structure on *S*. *mutans* biofilm. The exposure to blue light caused a reduction in biofilm development with a significant decrease of EPS production, which it is confirmed by the images, as presented in Fig 3. There was a visual difference between the blue light group (Fig 3A) and the control groups (Fig 3B and 3C).

**Fig 4:**
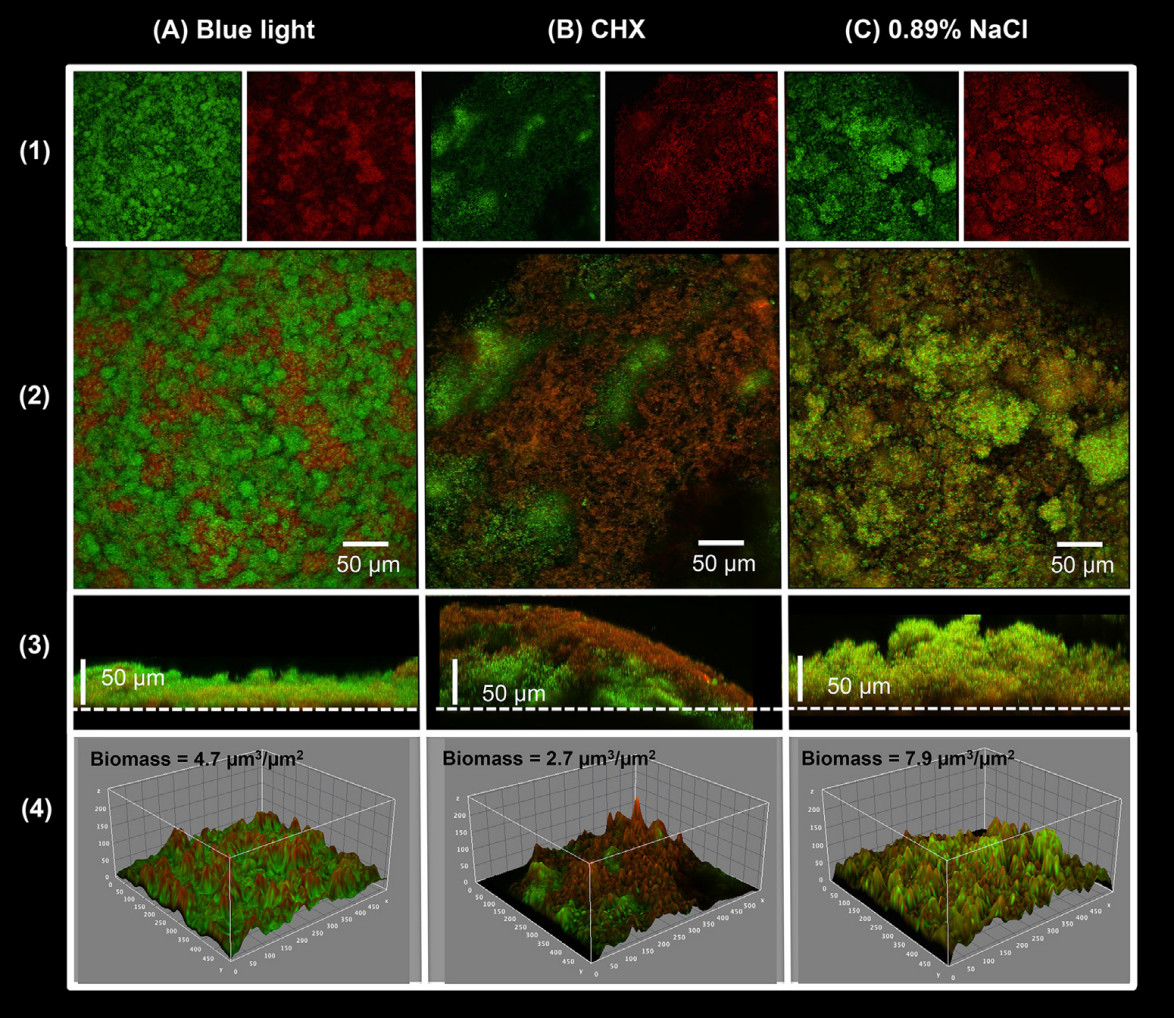
Viability and spatial arrangement in the *S*. *mutans* biofilms showed by confocal laser scanning microscopy. Five (5) day-old biofilms were stained with BacLight LIVE/DEAD and processed for CSLM. (A) Biofilm after the twice-daily blue light treatment during 5 days. (B) Biofilm after twice-daily treatment with 0.12% Chlorhexidine (positive control). (C) Biofilm after twice-daily treatment with 0.89% NaCl (negative control). (1) Red cells are considered dead and green cells are alive. (2) Overlap of live and dead stacks. (3) Orthogonal view of the overlap of live and dead stacks; dots line represents the interface biofilm/ HA substrate. (4) Representative three-dimensional images of the structural organization of the biofilms: rendered images of the outer layers of biofilms. The total bacterial biomasses calculated by COMSTAT are shown.

Confocal scanning laser microscopy (CSLM) representative images of bacteria in biofilms after twice-daily blue light exposure are shown in Fig 4. Multidimensional imaging of live (green) and dead (red) bacteria can be observed at different depths of S. mutans biofilm. The orthogonal view of biofilms showed that the biofilms treated twice-daily with blue light during their development are about 2-fold smaller than the controls. The total biomass of confocal images calculated by COMSTAT analysis confirm that the treatment with blue light is effective.

## Discussion

Light is an essential environmental cue for various organisms [23] Light is the major source of energy in the biosphere, and an essential signal that controls growth, development, and behavior of many different physiological mechanisms in most organisms. Long-term experience with phototherapy for the treatment of jaundice, cancer, and dermatological conditions has demonstrated its safety as well as its effectiveness [24]. A common example of the use of light as an antimicrobial is the PACT, which employs the combination of visible light of specific wavelengths and light-absorbing photosensitizers. Like PACT, blue light has emerged as a safe, low cost and effective antimicrobial therapy with little risk of antibiotic resistance [23].

Even though the use of PACT as an antimicrobial therapy has been suggested as an alternative method for oral biofilm treatment [9,16,25–28], this method has several limitations described in the literature. In addition to the non-selective antimicrobial characteristics of PACT, the photosensitizers have difficulty penetrating to the depths of the biofilm, resulting in less effectiveness on bacteria in biofilms than on bacteria in the planktonic phase [29].

The present study demonstrates that twice-daily blue light treatment, without a photosensitizer, applied twice-daily during *S*. *mutans* biofilm development remarkably inhibited the production of EPS-insoluble, which are responsible for the scaffold of the extracellular biofilm matrix. By reducing EPS- insoluble, blue light prevented biofilm development, with a minimal antimicrobial effect. In fact, it inhibited EPS-insoluble more effectively than the twice-daily application of the ‘gold-standard’ 0.12% CHX. Although there are more dynamic multispecies biofilm models that have been developed to facilitate investigation of antimicrobial agents in relation to the bacterial interactions [30,31], we have used an *S*. *mutans* model, a well-established model, to prove our concept. This model is ideal to study the polysaccharide matrix, the synthesis of extracellular polymeric substance (EPS) on surfaces, and the assembly of an insoluble matrix, all of which are critical for the existence of cariogenic bofilms [32]. *S*. *mutans* does not always dominate within dental plaque, but it is recognized that glucosyltransferases (Gtfs) from *S*. *mutans* play critical roles in the development of virulent dental plaque. These Gtf genes, among other functions, are responsible for producing the soluble and insoluble polysaccharides matrix [2]. As is reported in a series of studies, data collected *in vitro* using a model similar to *S*. *mutans* [21,33] correlates well with the data found in the *in situ* study [34].

Previous studies have demonstrated the cytotoxic effects of blue light [35]. Blue light wavelengths (400–500 nm) commonly used in dentistry for polymerization of composite resin restorations has been proposed as a direct means of affecting local bacterial infections [4,10,36]. Our results demonstrate a significant effect of this wavelength of blue light without a PS on *S*. *mutans* biofilms. Other studies have observed the bactericidal effect of blue light therapy on methicillin-resistant *Staphylococcus aureus* [37,38], *Staphylococcus aureus* [13], *Escherichia coli* [13], *Porphyromonas gingivalis* and *Fusobacterium nucleatum* [10]. Paschoal *et al*. [16] observed a complete inactivation of *S*. *mutans* in planktonic culture using the same light source and energy density, though a single application of blue light combined with curcumin as a photosensitizer was used at the end of the experimental period. It is well known that bacteria in biofilm are less sensitive to antibacterial agents than planktonic bacteria. Moreover, in the same study, a single does of blue light without PS, did not kill *S*. *mutans* [16]. Similar results were found by other authors [39], who actually state that a higher energy density of blue light is necessary to kill periodontal pathogens in planktonic cultures than in biofilm.

The extracellular polysaccharide matrices produced by *S*. *mutans* have been identified as the main factor responsible for the partial effectiveness of PACT. In the present study, daily exposure of biofilm to blue light decreased the levels of EPS-soluble when compared to the negative control. It has been established that the EPS-soluble may be readily digested and used as a reserve source of energy and contribute in part at least to the low pH values observed in cariogenic plaque [2,3]. On the other hand, EPS-insoluble levels produced under blue light treatment were not only significantly lower than those seen in the negative control group, but also lower than those seen with twice-daily treatment with the ‘gold-standard’ CHX. The EPS-insoluble plays a significant role on *S*. *mutans* adhesion and accumulation on the tooth surface [2]. In addition, it potentially changes the biofilm structure, resulting in increased porosity [40], which allows fermentable substrates to diffuse and to be metabolized in the deepest parts of the biofilm [41]. According to a previous study [6], the formation of the specific EPS domains could be explained by the presence of enzymatically active Gtfs, in which gtfC has greatest affinity for HA discs whereas gtfB binds preferentially to bacterial surfaces [42]. Exposure of *S*. *mutans* to blue light might indicate that there is a decreased expression of gtfs, considering a significantly reduced biomass and bacterial aggregates and more disturbances in the development and architecture of the *S*. *mutans* biofilm. This hypothesis is under investigation.

According to Dai [12], blue light can be sensed by numerous bacteria and can induce physiological responses elicited by the blue light receptors; as a result, blue light can regulate bacterial motility, suppress biofilm development, and potentiate light inactivation of bacteria. According to Gomelsky [31], there are three different classes of blue-light receptors in the bacteria: light, oxygen, and voltage (LOV) domains (flavins), blue light photoreceptor (BLUF) domains (flavins), among others. These receptors are linked to GGDEF and EAL protein domains, which are involved in synthesis and hydrolysis, respectively, of cyclic di-GMP (c-di-GMP)–a ubiquitous second messenger specific to bacteria. The c-di-GMP has been shown to inhibit flagella-based motility and to stimulate synthesis and/or excretion of biofilm matrix components. GGDEF and EAL domains are also involved in light-dependent regulation of biofilms and motility, inducing biofilm inhibition [43].

To determine the EPS matrix morphology of *S*. *mutans* biofilms with respect to topography, we used VPSEM to accurately image the EPS matrix [20]. In our study, the *S*. *mutans* biofilm topography was visibly decreased after blue light irradiation, in both CSLM and VPSEM images. This result suggests that blue light might have promoted disorganization and disaggregation of the microorganisms in the biofilm, inhibiting their growth and metabolism, consistent with other studies of the effect of blue light on the metabolism and viability of *S*. *mutans* in biofilm [4,14]. It is important to note that the main mechanism by which blue light reduce the biofilm development is by inhibiting the polysaccharide matrix formation, whereas CLSM gives us information regarding bacterial biomass.

Furthermore, according to Feuerstein [5], absorbed light at different wavelengths inhibits bacterial growth by two main mechanisms: one photochemical and one photothermal. The photochemical mechanism is based on the formation of reactive oxygen species (ROS). It was considered that in the absence of exogenous photosensitizers, visible light primarily affects porphyrin-containing black-pigmented oral bacteria, such as *Porphyromonas gingivalis*. Non-coherent blue-light sources such as the halogen lamp, LED and the plasma arc provide a phototoxic effect, mainly on planktonic cultures of the Gram-negative bacteria *Porphyromonas gingivalis* and *Fusobacterium nucleatum* associated with periodontal diseases [36]. However, doses of blue light must be at least seven to ten times higher in order to kill Gram-positive *S*. *mutans* and *Enterococcus faecalis*, demonstrating a minimal antimicrobial effect against these two species [36]. Light regulates the production of EPS in *R*. *leguminosarum* biofilms, and LOV-domain histidine kinase (LOV-HK), suggesting that a light sensor is involved in this process [44]. For another soil bacteria, *Caulobacter crescentus*, blue light has also been associated with cell-cell and cell-surface attachment [45], suggesting that blue light helps regulate the bacterial surface. In contrast to some soil bacteria, it is unclear how blue light specifically affects the physiology of *S*. *mutans* and by which mechanism(s). The wavelengths of blue light are highly absorbed by endogenous bacterial photosensitizers such as flavins and cytochromes, resulting in a phototoxic effect on the bacteria, mediated by ROS formation. Hence, the damaged cells may became more vulnerable to oxygen in the air when re-organizing into the new biofilm; thus, this effect appeared mostly in the outer layers [46].

The energy density utilized in the present study was based on a pilot study conducted in our laboratory (data not published). To our knowledge, this is the first report of twice-daily use of blue light to prevent oral biofilm development. This exposure time prevented *S*. *mutans* biofilm development by reducing the production of EPS-insoluble. Future study is needed to establish the parameters of blue light exposure that minimizes polysaccharide while remaining non-toxic to the oral tissues.

Therefore, we conclude that twice-daily treatment prevented the *S*. *mutans in vitro* biofilm matrix development, being more effective in reducing the production of EPS-insoluble than the ‘gold-standard’ anti-plaque 0.12% chlorhexidine. This leads us to believe blue light is a promising therapeutic approach for matrix-rich biofilm prevention, such as the cariogenic biofilm, by interfering with one of its most relevant virulence factors: the extracellular polysaccharide matrix. This work generates new hypotheses, and may have importance beyond the oral cavity, as polysaccharide matrices are present in all medically important biofilms.
